# Improvement of Presbyopia, Dry Eye, Intraocular Pressure, and Near Vision Through Cassiae Tea Consumption

**DOI:** 10.3390/medicina61010035

**Published:** 2024-12-29

**Authors:** Mei Fan, Jen-Lin Hung, Shao-Huan Hung, Li-Chai Chen, Chi-Ting Horng

**Affiliations:** 1 Department of Pharmacy, Kaohsiung Armed Forces General Hospital, Kaohsiung 802, Taiwan; 2 Master of Science Program in Health Care, Department of Nursing, Meiho University, Pingtung 912, Taiwan; 3 Department of Pharmacy, Tajen University, Pingtung 907, Taiwan; 4 Department of Ophthalmology, Kaohsiung Armed Forces General Hospital, Kaohsiung 802, Taiwan

**Keywords:** cassiae tea, presbyopia, pupil size, dry eye, glaucoma, smartphone, smartphone presbyopia

## Abstract

*Background and Objectives*: This study aimed to illustrate a novel method for improving presbyopia by drinking cassiae tea. *Materials and Methods*: A total of 425 eyes from 425 participants (aged 52.5 ± 9.5 years) were recruited and divided into several experimental groups over a 6-month period. Participants consumed cassiae tea daily (10 g of cassiae semen brewed in 500 cc of water). Meanwhile, control group participants consumed 500 cc of plain water along with 1000 mg of vitamin C each day. Experiments 1 and 2: Participants with severe dry eye and intraocular pressure (IOP) > 30 mmHg were enrolled, and outcomes were assessed for these conditions, respectively. Experiments 3, 4, and 7: These experiments evaluated pupil size, near vision, and serum superoxide dismutase (SOD) levels in two groups of volunteers. Experiment 5: Different quantities of cassiae tea were prescribed to various groups, and near vision was measured. Experiment 6: Three questionnaires assessed presbyopic symptoms after cassiae tea consumption. Experiment 8: The antioxidant activity of cassiae tea compared with other bioactive compounds and Chinese herbs was evaluated using the DPPH test. *Results*: By the fourth month of the study, participants experienced increased tear volume and reduced IOP. Pupil size constricted, near vision improved, and serum SOD levels increased. Furthermore, greater consumption of cassiae tea was correlated with better near vision. The antioxidant activity of cassiae tea was found to surpass that of anthocyanins, wolfberry, and vitamin C. *Conclusions*: Drinking cassiae tea improves dry eye symptoms, reduces IOP, regulates pupil size, and enhances near vision due to its excellent antioxidant and pharmacological properties. These benefits may particularly aid individuals with presbyopia and those engaged in near-distance activities, such as smartphone use.

## 1. Introduction

Presbyopia is a prevalent eye disorder characterized by poor ocular accommodation, resulting in difficulty focusing on near objects. It is considered one of the common diseases of civilization. Recent research shows that presbyopia not only affects near vision (approximately 20–40 cm, such as for smartphone use) but also impacts intermediate vision (50–100 cm, for desktop computer use). “Near work” is defined as any activity requiring focus on objects within arm’s reach (approximately 40 cm or closer). The global prevalence of presbyopia is growing. In 2015, it affected 1.8 billion people worldwide, and this number is predicted to rise to 2.1 billion by 2030 [1]. The prevalence and severity of presbyopia increase with age, with 30% of the population in their mid-to-late 40s experiencing symptoms. Furthermore, presbyopia often co-occurs with dry eye and glaucoma, conditions that are commonly seen in individuals with presbyopia and smartphone users. In clinical practice, patients with presbyopia are often affected by related issues such as dry eye and glaucoma. Interestingly, adults usually begin to notice symptoms of presbyopia and dry eye around age 40. The prevalence of glaucoma is 1.0% in people aged 40–44, but this increases significantly to 3.6% in those aged 60–64.

The inability of middle-aged individuals to focus on near objects has been a concern since as early as the 4th century BC. Today, presbyopia—and even early presbyopia—has become a significant social issue, impairing near vision and interfering with personal and professional tasks, such as operating smartphones and portable computers [2]. Those with presbyopia often experience difficulty performing near-vision-related activities. Common symptoms include eyestrain, ocular fatigue, blurred vision in low-light conditions, and headaches during near-field work. These symptoms are particularly prominent in office workers, drivers, computer engineers, and teachers [3]. Presbyopia is a well-recognized condition because of its significant impact on quality of life. It can lead to sleep disturbances, reduced productivity, and diminished economic contributions [4]. Additionally, individuals with presbyopia may experience interruptions to daily activities and suffer from anxiety or depressive moods [5]. Thus, strategies for managing presbyopia are critical for public ocular health.

In 2015, an estimated 1.8 billion people were affected by presbyopia globally, with 826 million of them having no or inadequate vision correction [6]. When viewing near objects, the ciliary muscles contract to release zonular tension, allowing the crystalline lens to round and focus. With presbyopia, the ability to accommodate decreases due to weakening of the ciliary muscle-lens zonule apparatus and lenticular sclerosis. The concept of “smartphone presbyopia” has emerged alongside the widespread use of digital devices. By 2024, the number of smartphone users worldwide surpassed 4 billion. Prolonged use of smartphones at close distances can paralyze the adjustment mechanisms of the ciliary muscles and zonular fibers. According to Wang’s study, 37.8% of smartphone users experience presbyopia, and 64.3% of individuals with presbyopia report difficulty using their smartphones [7]. Excessive smartphone use is also associated with conditions such as dry eye, poor head and neck posture, and periocular strain caused by increased intraocular pressure (IOP). Smartphone presbyopia develops when devices are held close to the eyes for extended periods. Historically, presbyopia was considered a condition of the elderly. However, younger individuals aged 20–30, and even junior high school students, are increasingly showing signs of presbyopia due to frequent smartphone use. Smartphone addiction has also been linked to emotional anxiety, obsessive-compulsive behavior, and diminished social skills, which contribute to both physical and psychological disorders.

As a result, physicians are now paying greater attention to the adverse effects of smartphone-induced presbyopia to promote overall health [8]. Presbyopia can be managed through several methods, including spectacles, contact lenses, surgery, and eye drops. Reading glasses are the most common solution, typically prescribed to individuals over 40 for close activities. In developed countries, 62% of individuals with presbyopia use reading glasses, compared to only 26% in low-income countries. However, spectacle prescriptions may require regular adjustments, and some individuals find it inconvenient to frequently put on and remove their glasses in different situations [9]. Bifocal and multifocal contact lenses provide an alternative for those seeking glasses-free solutions, as they offer stereopsis and binocular vision. However, wearing contact lenses carries certain risks, and some individuals experience vertigo or dizziness when using multifocal lenses. Surgical options, such as PresbyLASIK, corneal inlays, conductive keratoplasty, and scleral expansion, offer more permanent solutions. However, these procedures involve irreversible tissue changes, which can make patients hesitant [10]. Non-invasive pharmacological treatments, such as eye drops, are still being explored. For example, Benozzi and colleagues developed an eye drop combining pilocarpine and diclofenac (NSAIDs) [11], while Renna et al. proposed a formulation containing pilocarpine, nepafenac (NSAID), and naphazoline [12]. However, pilocarpine can cause side effects like dizziness, nausea, sweating, drowsiness, weakness, eye stinging, and facial flushing, which discourage its use [13]. Moreover, pharmacotherapy for presbyopia is not yet widespread due to limited clinical trials and test subjects. In Chinese society, the tradition of drinking tea has evolved into a deeply ingrained cultural practice. Many herbal teas, including cassia tea, are valued for their health benefits. Cassia tea is made from cassiae semen (cassia seeds), also known as Jue Ming Zi. It has been consumed for centuries and remains a popular remedy in traditional Chinese medicine (TCM) [14]. Cassia seeds grow in various countries, including India, Pakistan, Singapore, and Bangladesh, and cassia tea is also widely consumed in Japan and Korea as a health beverage [15]. Research suggests that cassiae semen may help treat conditions such as constipation, Parkinson’s disease, eye disorders (e.g., ocular tearing, red eye, night blindness, and retinopathy), cardiovascular disease, liver disorders, hyperlipidemia, diabetes mellitus (DM), and Alzheimer’s disease [16,17].

In our study, we aimed to investigate the clinical effects of Cassiae tea on presbyopia, along with associated issues such as dry eye, glaucoma, and accommodative insufficiency, particularly in middle-aged individuals and near-distance workers, such as smartphone users.

## 2. Materials and Methods

### 2.1. Participants

This study comprised a series of experiments aimed at evaluating the effects of cassiae tea on lowering intraocular pressure (IOP), alleviating dry eye symptoms, adjusting pupil size, and improving near vision. A total of 425 eyes from 425 volunteers were enrolled. The study was a controlled, double-blind, randomized trial conducted over six months, with participants instructed to attend regular visits at monthly intervals. Written informed consent was obtained from all participants, and the study was approved by the Institutional Review Board of Kaohsiung Armed Forces General Hospital (approval number: KAFGHIRB 109-019). Additionally, experiments 6 and 7 were conducted in our medical laboratory. All experiments took place between March and September 2021.

### 2.2. Preparation of Cassiae Semen

Based on previous literature, the median daily total fluid intake was approximately 1488 mL [18]. The safe oral dosage of cassiae tea was determined following Huang’s recommendations [19], and we consulted with several traditional Chinese medicine (TCM) practitioners in Taiwan for further guidance. Different doses of cassiae tea were prepared for the study. Each cup of cassiae tea was made with 5 g of fried cassia seeds steeped in 250 mL of hot water (70 °C). Accordingly, two cups of cassiae tea (500 mL) contained 10 g of cassia seeds, while three cups (750 mL) contained 15 g of cassia seeds. Participants in the control group consumed 1000 mg of vitamin C dissolved in 500 mL of plain water daily, as outlined in Zychowska’s study [20].

### 2.3. Ocular Examinations

**Figure 1:**
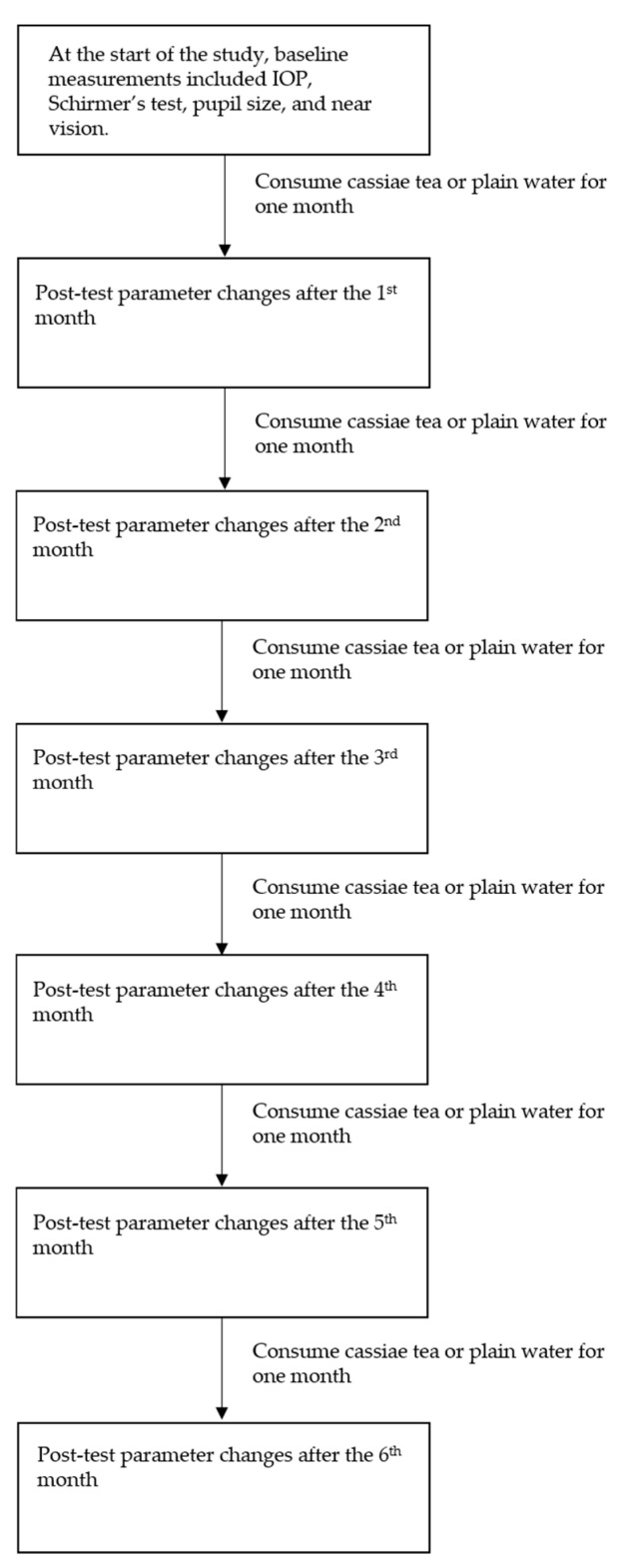
Diagrams for experiments 1, 2, 3, 4, and 6, with various examinations conducted monthly.

**Figure 2:**
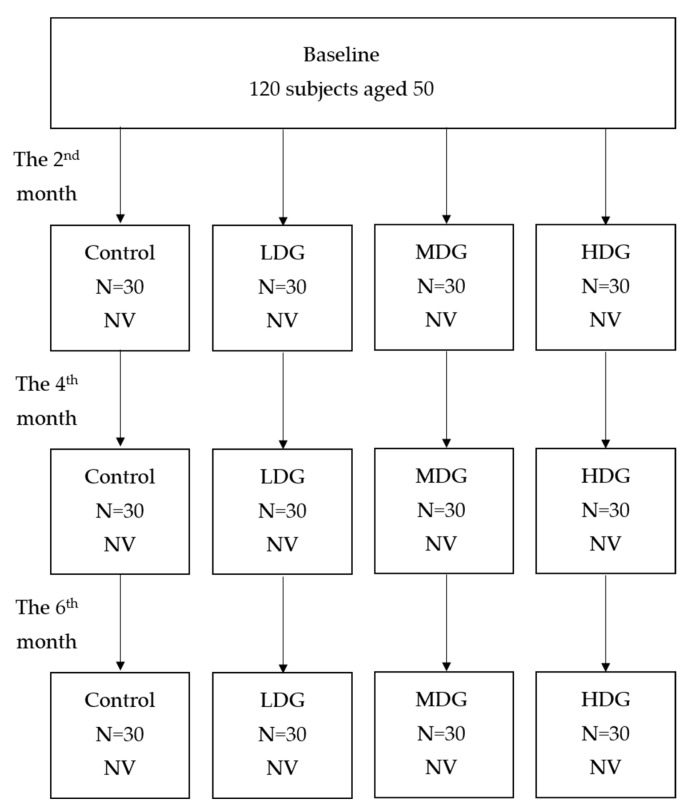
A total of 120 participants aged 50 were divided into four groups: control, LDG, MDG, and HDG, based on different doses of cassiae tea. Near vision (NV) was assessed bi-monthly.

**Figure 3:**
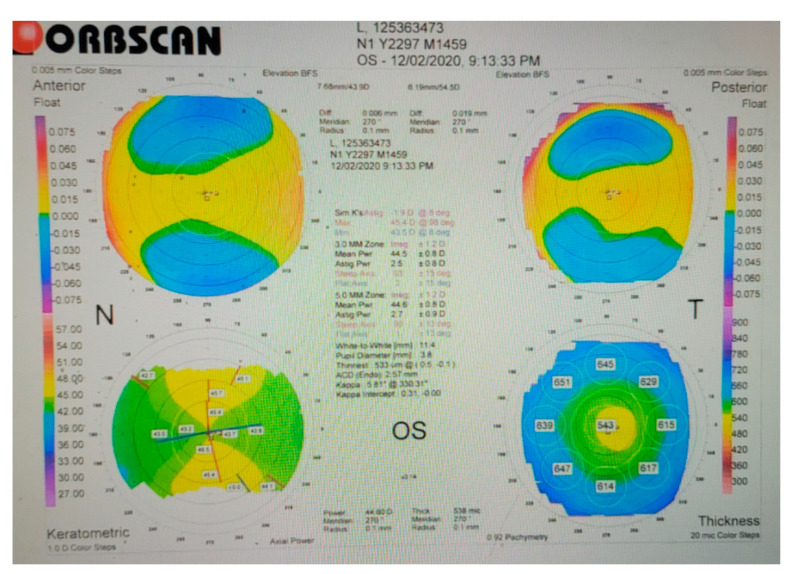
Pupil sizes were measured using Orbscan corneal topography.

At the beginning of the study, baseline ocular parameters were measured, including IOP, Schirmer’s test scores, pupil size, and near vision. Participants then received their assigned treatments over a six-month period, with changes in these parameters assessed monthly. At the end of the study, the measured values were compared to baseline. The flowcharts for experiments 1, 2, 3, 4, and 6 are presented in Figure 1, while the procedure for experiment 5 is shown in Figure 2. In addition to the primary parameters, other ocular examinations were performed using a slit-lamp microscope (SL-D701, Topcon, Itabashi, Japan), a pneumotonometer (NT-530/510, Nidek, Gamagori, Japan), and non-mydriatic retinal photography (CR-2 AF, Canon, Tokyo, Japan). These examinations assessed the cornea, lens, anterior chamber (A/C), IOP, optic nerve, and retinal morphology. Pupil size was evaluated using an Orbscan Corneal II Topographer (Bausch and Lomb, Bridgewater, NJ, USA) (Figure 3) [21]. The values of IOP, Schirmer test scores, pupil size, and other ocular parameters were all assessed by a single well-trained technician to minimize personal bias. Throughout the six-month study period, the symptoms and signs of each participant were closely monitored and documented.

### 2.4. Inclusion/Exclusion Criteria

Volunteers who had not undergone intraocular operations, such as cataract surgery, trabeculectomy, pars plana vitrectomy, various ocular laser therapies, or any refractive surgeries within the past two years, were included in the study. However, individuals with diabetes mellitus (DM), hypertension, autoimmune diseases (e.g., systemic lupus erythematosus, ankylosing spondylitis), or ocular trauma were excluded to minimize experimental bias.

### 2.5. Experiment 1: Evaluation of Dry Eye (Total Participants = 60)

Schirmer’s test, a relatively simple, rapid, and efficient method for diagnosing dry eye, was used to assess tear production by measuring baseline and reflex tear secretion [22]. This test has a sensitivity of 92% and a specificity of 88%. A total of 60 participants with severe dry eye (test score < 5 mm/5 min) were recruited and divided into two groups: control (n = 30) and test (n = 30). The test group consumed two cups (500 cc/day) of cassiae tea, while the control group consumed 1000 mg of vitamin C dissolved in 500 cc of water daily. Schirmer’s test strips (Schirmer strips, Win Way Technology Co., Ltd., Kaohsiung, Taiwan) were placed in each participant’s inferior conjunctival fornix without topical anesthesia, and the moisture content was recorded after 5 min. A wet filter paper length of >10 mm was considered normal, while <5 mm indicated severe dry eye. Changes in test scores were monitored monthly over six months to evaluate whether cassiae tea alleviated dry eye symptoms.

### 2.6. Experiment 2: Assessment of IOP (Total Volunteers = 45)

Reducing intraocular pressure (IOP) is critical for managing glaucoma and ocular hypertension. Ocular hypertension is defined as IOP > 21 mmHg without vision loss, optic disc damage, or visual field defects. Poor IOP control increases the risk of glaucoma progression [23]. In this experiment, 45 participants with ocular hypertension were divided into three groups: control (15 participants consuming 500 cc/day of vitamin C water), test (15 participants consuming two cups/day of cassiae tea), and eye drop (15 participants using brimonidine [Alphagan^®^, Allergan, CA, USA], an α2-adrenergic agonist, twice daily) [24,25]. IOP was measured three times for each participant using an air-puff pneumotonometer, and the average value was recorded. IOP values were assessed monthly to determine whether cassiae tea effectively reduced IOP levels.

### 2.7. Experiment 3: Pupil Size Assessment (Total Subjects = 60)

A total of 60 participants were divided into two groups: control (n = 30) and test (n = 30, consuming two cups/day of cassiae tea for six months). Monthly pupil size measurements were performed using Orbscan topography. Horizontal diameters were standardized for all measurements to account for the non-circular shape of human pupils [26]. This experiment aimed to evaluate whether cassiae tea affected pupil size over time.

### 2.8. Experiment 4: Near Vision Assessment with the Same Dose of Cassiae Tea (Total Participants = 60)

To analyze the effects of a consistent dose of cassiae tea on near vision, 30 participants in the control group and 30 in the test group (consuming two cups/day of cassiae tea) were evaluated. Near vision was assessed using the Jaeger eye chart (Rosenbaum Pocket Eye Chart) placed 40 cm in front of participants under illuminated conditions [27]. Moreover, all participants had their near vision tested under adequate room lighting of approximately 500 lux to ensure clear visibility of the Jaeger chart. Near vision was expressed as “J” values. The study aimed to determine whether consuming cassiae tea improved near vision over six months.

### 2.9. Experiment 5: Near Vision Assessment with Different Doses of Cassiae Tea (Total Volunteers = 120)

A total of 120 participants were randomly assigned to four groups: a control group (consuming 1000 mg of vitamin C in 500 cc water daily), a low-dose group (LDG, consuming one cup/day of cassiae tea), a middle-dose group (MDG, consuming two cups/day), and a high-dose group (HDG, consuming three cups/day). Near vision was assessed at baseline, the 2nd, 4th, and 6th months using Scheffé’s test. Additionally, William’s test was employed to compare near vision changes across the four groups at the three time points. The results were expressed as “J” values to evaluate the pharmacological effects of different cassiae tea doses on near vision.

### 2.10. Experiment 6: Visual Quality Questionnaire After Drinking Cassiae Tea (Total Participants = 135)

To assess subjective visual experiences, a questionnaire adapted from Shirnesshan et al. [28] was administered to participants for experiments 1–5. Three key questions were included:

1. Did you experience any ocular discomfort (e.g., irritation, dryness, or foreign body sensation) after drinking cassiae tea?
2. Did you find it difficult to observe near objects or read after consuming cassiae tea?
3. Did you experience difficulty performing near tasks after consuming cassiae tea? The questionnaire was completed at baseline, the 2nd, 4th, and 6th months.

### 2.11. Experiment 7: Superoxide Dismutase (SOD) Values (Total Participants = 80)

Eighty participants were divided into two groups: test (n = 40, consuming two cups/day of cassiae tea) and control (n = 40). Blood samples (5 cc venous blood) were collected at baseline and after six months. Samples were centrifuged at 5000 rpm for 5 min to separate serum, which was stored at 30 °C for analysis. SOD levels, measured in U/mL, were determined using spectrophotometric assays. SOD values are commonly used as a biomarker for oxidative stress in eye diseases, and this experiment aimed to evaluate the impact of cassiae tea on SOD levels [29].

### 2.12. Experiment 8: Total Antioxidant Abilities of Cassiae Tea and Other Biochemical Substances

The DPPH method was used to assess the antioxidant properties of cassiae semen, wolfberry, astaxanthin, anthocyanins, and vitamin C. DPPH (2,2-diphenyl-1-picrylhydrazyl) is a stable free radical commonly used in antioxidant studies [30]. Test materials were prepared at concentrations of 1, 5, 10, 20, and 40 mg/mL in methanol. Samples were mixed with Tris-HCl buffer and DPPH-ethanol solution and incubated for 30 min. Absorbance at 517 nm was measured to calculate the scavenging effect (%) using the following equation: Inhibition (%) = [(Absorbance of Control − Absorbance of Test Sample)/ Absorbance of Control] × 100.The results were plotted with concentration on the *x*-axis and scavenging effect (%) on the *y*-axis [31].

### 2.13. Statistical Analysis

Schirmer’s test scores, IOP values, pupil sizes, near vision measurements, and SOD levels were expressed as the mean ± standard deviation (SD). Data were analyzed using SPSS SAS version 25 (SAS Inst., Cary, NC, USA). Scheffé’s method was used for comparisons with baseline values at the 2nd, 4th, and 6th months (experiments 1, 3, 4, 5, and 6), while one-way analysis of variance (ANOVA) was employed in experiment 2. For experiment 5, William’s test was used to evaluate dose-dependent effects. Chi-square (Χ^2^) tests were performed to compare proportions [32]. A *p*-value of <0.05 was considered statistically significant.

## 3. Results

The characteristics of the participants in our experiments included 223 males and 202 females, with 213 right eyes and 212 left eyes evaluated. The mean age of the participants was 52.5 ± 9.5 years. All participants completed the experiments without withdrawal. Additionally, no significant damage to the cornea, lens, vitreous cavity, retina, or optic nerve was observed. Furthermore, no notable adverse events such as diarrhea, gastrointestinal disorders, insomnia, irritability, ocular pain, skin rash, or accommodation-induced headaches were reported after consuming cassiae tea.

### 3.1. Experiment 1 (Table 1)

The Schirmer’s test scores in the test group showed a significant increase starting from the 4th month (15.2 ± 0.8 mm, *p* < 0.05) and continued to improve by the 6th month (17.6 ± 0.9 mm, *p* < 0.05). By contrast, the scores in the control group remained unchanged (*p* > 0.05). These results demonstrate that cassiae tea supplementation effectively enhanced tear secretion from the lacrimal glands, as indicated by the increased test scores. Therefore, incorporating cassiae tea into a regular diet may be beneficial for managing dry eye symptoms.

### 3.2. Experiment 2 (Table 2)

Participants with elevated intraocular pressure (IOP) were treated with cassiae tea and topical application of Alphagan^®^. The results showed a significant reduction in IOP, from approximately 31 mmHg to 20 mmHg, starting from the fourth month of treatment (*p* < 0.05). This suggests that cassiae tea has a comparable IOP-lowering effect to Alphagan^®^. By contrast, participants in the control group exhibited no significant changes in IOP (*p* > 0.05). Therefore, we conclude that consuming cassiae tea may effectively reduce IOP levels in cases of glaucoma and ocular hypertension.

### 3.3. Experiment 3 (Table 3)

Pupil sizes in the control group did not show any significant changes (*p* > 0.05). However, the pupil diameters in the test group, which consumed 500 cc/day of cassiae tea, exhibited a noticeable constriction starting from the 4th month (2.4 ± 0.4 mm, *p* < 0.05) and persisting until the 6th month (2.3 ± 0.3 mm, *p* < 0.05). According to these findings, we propose that sufficient intake of cassiae tea may gradually lead to smaller pupil sizes.

### 3.4. Experiment 4 (Table 4)

No significant changes in near vision were observed in the control group (*p* > 0.05). However, in the test group, near vision improved noticeably by the 4th month (J 3.3 ± 1.2, *p* < 0.05) and continued to improve by the 6th month (J 2.5 ± 0.8, *p* < 0.05). Effective presbyopic correction is commonly defined as near vision at 40 cm, measured with habitual distance refractive correction, showing an improvement of at least one line on the Jaeger eye chart [33].

For example, in this study, near vision improved from J5.4 at baseline to J3.3 in the 4th month and further to J2.5 in the 6th month after drinking cassiae tea. Therefore, we concluded that cassiae tea significantly enhances near vision in consumers.

### 3.5. Experiment 5 (Table 5)

No significant changes in near vision were observed in the control group (*p* > 0.05). However, in the test group, improved near vision was detected starting from the 4th month (J 3.3 ± 1.2, * *p* < 0.05) and continued through the 6th month (J 2.5 ± 0.8, * *p* < 0.05). Effective presbyopic correction is commonly defined as improved near vision at 40 cm with habitual distance refractive correction, showing an improvement of at least one line on the Jaeger eye chart [33]. For example, near vision improved from J 5.4 (baseline) to J 3.3 (4th month) and J 2.5 (6th month) after consuming cassiae tea. Therefore, we conclude that cassiae tea significantly enhances consumers’ near vision.

### 3.6. Experiment 6 (Figure 4)

The most noticeably affected activities included writing, sorting grains, trimming fingernails, recognizing small objects, and threading a needle. At the beginning of the experiment, approximately 85% of participants reported ocular discomfort, difficulty seeing objects, and challenges performing near-distance tasks. After consuming 2 cups/day of cassiae tea, presbyopic symptoms gradually improved. By the end of Experiment 6, the percentage of emotional and physical problems caused by presbyopia had reduced to 28–40%. Thus, we confirmed that cassiae tea consumption could assist individuals with presbyopia in improving near-distance performance, such as using smartphones.

### 3.7. Experiment 7 (Table 6)

We confirmed that serum SOD levels significantly increased starting from the 4th month (*p* < 0.05). By contrast, no noticeable changes were observed in the control group (*p* > 0.05). According to these findings, we recommend adequate cassiae tea supplementation to enhance serum SOD levels, which play a crucial role in antioxidant activity. This improvement may support better ocular accommodation and alleviate presbyopic symptoms [34].

### 3.8. Experiment 8 (Figure 5)

In this experiment, we used methanol at a concentration of 10 mg/mL as an example to evaluate the free radical scavenging effects. The results showed the following order: astaxanthin > cassiae semen > wolfberry > anthocyanin > vitamin C (control) (Figure 5). In other words, the antioxidant activity of cassiae semen was higher than that of many health supplements (e.g., anthocyanin and vitamin C) and Chinese herbal medicines (e.g., wolfberry). Numerous studies have demonstrated that astaxanthin exhibits superior antioxidant properties [35]. To our surprise, the antioxidant activity of cassiae semen was only slightly lower than that of astaxanthin. According to these findings, we strongly recommend cassiae tea as a beneficial option for managing oxidative stress associated with eye conditions such as presbyopia, dry eye, glaucoma, ocular hypertension, and reduced accommodative ability.

## 4. Discussion

Presbyopia is an age-related near-vision impairment commonly affecting individuals aged 40 years and older. The prevalence of presbyopia varies across countries, ranging from 48% to 55%, and increases with age. Current global analyses estimate that approximately 1.1 billion people are impacted by presbyopia, with one-third being 50 years or older [36]. From a physiological and pathological perspective, presbyopia is primarily an inevitable condition, and its prevalence within a population correlates with the percentage of individuals surviving to older age. Symptoms of presbyopia typically begin between 42 and 44 years of age, with complete loss of accommodation occurring between the ages of 50 and 55, often necessitating the use of corrective measures such as reading glasses.

Numerous risk factors are associated with presbyopia, including aging, occupational exposure, prior intraocular surgeries, fatigue, exposure to hair dye, certain medications (e.g., antihistamines, antidepressants, antipsychotics, and diuretics), diabetes mellitus (DM), multiple sclerosis, cardiovascular diseases, anemia, smoking, glaucoma, thyroid conditions, and higher education levels [37]. In recent years, impaired near vision due to prolonged use of mobile computers and smartphones has become a significant concern. Adequate correction of presbyopia is essential in today’s society to prevent social limitations and negative impacts on daily living activities, work performance, career opportunities, and self-esteem [38]. Addressing the promotion of senile presbyopia and “smartphone presbyopia” has thus become a global public health priority.

Uncorrected presbyopia can result in symptoms such as asthenopia, periocular soreness, and headaches. It can also hinder daily activities; for example, users of portable computers and smartphones may struggle to see near text clearly. Notably, some individuals experience presbyopic symptoms as early as their 20s or 30s, a phenomenon referred to as “premature presbyopia” or “smartphone presbyopia” [39]. Excessive use of smartphones, tablets, and video display terminals—defined as more than four continuous hours per day—can lead to accommodative paralysis and early development of presbyopia [40]. With smartphone ownership exceeding 60% worldwide, prolonged screen exposure can exacerbate conditions like dry eye and ocular convergence insufficiency. Moreover, sustained accommodation due to prolonged viewing of small text may lead to intraocular pressure (IOP) elevation, creating a close link between presbyopia and glaucoma [41]. Reading on smartphones in dim lighting conditions can increase IOP by over 200%, potentially causing damage to the optic nerve region [42]. Therefore, healthcare professionals play a pivotal role in addressing and managing smartphone-related presbyopia.

Accommodation refers to the eye’s ability to adjust refractive power to achieve focused retinal images. The amplitude of accommodation (AA) is a quantitative measure of this ability and is crucial for maintaining clear vision at varying distances [43]. Presbyopia reflects a reduction in ocular accommodative range due to weakened ciliary muscles and progressive cataract formation. The condition is further exacerbated by increased stiffness of the crystalline lens caused by the deposition of damaged fibers and cross-linking of α-crystalline proteins [44].

Younger individuals typically have a higher AA level, which supports better vision due to stronger accommodative ability. Clinically, AA is measured in diopters (D), with individuals over 40 years of age requiring correction when AA falls below 5.00 D. Reading spectacles are the most common and convenient method of correction, with approximately 66% of adults with presbyopia in the United States and 68% in Britain using them in 2023. Options like bifocal and progressive lenses allow for better viewing of near objects, although some drawbacks persist, such as peripheral distortion and loss of depth perception [45]. Invasive treatments like conductive keratopathy and anterior ciliary sclerotomy carry risks of complications, including inflammation, infection, and elevated IOP, which can cause anxiety among patients [10].

Modern pharmacologic therapies for presbyopia, such as miotic agents like pilocarpine, offer a spectacle-free alternative. However, these treatments provide only temporary relief and may cause side effects, including nausea, runny nose, headaches, and even retinal detachment in severe cases [46,47]. Further studies with larger sample sizes are necessary to establish their safety and efficacy.

Complementary and alternative therapies, particularly herbal teas, have gained popularity in recent years. Cassiae semen, derived from the dry mature seeds of Cassia obtusifolia L. or Cassia tora L., has long been valued in traditional Chinese medicine (TCM) for its health benefits, including its effects on ocular health [48]. Commonly consumed as a tea with chrysanthemum flowers for added flavor, cassiae semen has been documented in historical texts for its ability to alleviate vertigo and headaches and enhance visual clarity. Modern research suggests that cassiae semen exhibits a range of pharmacological activities, such as promoting digestive and nervous system health and addressing bacterial and inflammatory conditions [49,50]. However, its specific effects on presbyopia remain unexplored. This study aimed to investigate its potential in managing presbyopia and related eye conditions.

### 4.1. Dry Eye Disease

The prevalence of dry eye disease (DED) ranges from 6% to 33% of the global population. In Taiwan, the prevalence of DED is estimated to be between 33.3% and 47.5% in ophthalmology outpatient clinics. The occurrence of DED increases with each passing decade of life starting at age 40, with the severity worsening as individuals age. Apart from aging, other risk factors for DED include being female, having certain autoimmune diseases, wearing contact lenses, taking specific medications, and living in certain environmental or geographic conditions [51].

DED is closely associated with presbyopia. Aging not only affects the ocular surface but also exacerbates an already imbalanced tear film, leading to dry eye symptoms. Long-term smartphone use is another significant contributor to DED. In 2018, 89.5% of South Korea’s population aged 3 and older used smartphones. According to Park’s study, overuse of digital screens—exceeding 4 h per day in near-field activities such as gaming, internet browsing, and smartphone use—significantly increases the risk of DED [52]. Moreover, smaller display sizes on smart devices also pose a risk for DED.

DED is primarily diagnosed using Schirmer’s test in clinical settings. Blinking plays a crucial role in maintaining ocular lubrication by discharging the meibomian and lacrimal glands, which provide essential fluids and lipids. On average, a person blinks 15 times per minute to maintain eye health. However, both blink amplitude and velocity decrease with age. Smartphone users, particularly during near work, exhibit a significantly reduced blink rate (8.9 blinks/minute), which correlates with a decrease in Schirmer’s test scores [53]. Middle-aged individuals often spend leisure time on smartphones, exacerbating dry eye problems, especially in those with presbyopia.

Since 2017, DED has been redefined as a multifactorial disease of the ocular surface, characterized by discomfort, visual disturbance, and reduced quality of life. DED is associated with tear film instability, hyperosmolarity, inflammation, neurosensory abnormalities, and loss of homeostasis. Without proper treatment, corneal cell apoptosis and goblet cell loss can occur [54]. Ocular surface inflammation caused by DED can lead to complications such as punctate epithelial keratitis, filamentary keratitis, or corneal ulcers.

Treatment options for DED include artificial tears, anti-inflammatory agents (e.g., corticosteroids), and antibiotics (e.g., tetracycline) to reduce inflammation. In our study, Schirmer’s test scores increased to 15.2 ± 0.8 mm at the 4th month and further to 17.6 ± 0.9 mm at the 6th month after consuming cassiae tea. The active polysaccharides and biochemical compounds in cassiae semen were shown to reduce inflammation and improve dry eye symptoms [55]. On the basis of these findings, we recommend an integrative approach to treating DED, combining modern artificial tears with daily consumption of cassiae tea.

### 4.2. Glaucoma and Ocular Hypertension

The onset of presbyopia and glaucoma typically occurs after age 40, and their relationship is significant. In 2020, approximately 76 million people worldwide were affected by glaucoma, with 8 million suffering from visual impairment or blindness. By 2040, this number is projected to rise to 111.8 million [56]. In Taiwan, over 340,000 people were affected by glaucoma in 2023. Alarmingly, 90% of glaucoma patients are unaware of their symptoms—such as halos around bright lights, periocular pain, and nausea—until they are properly diagnosed. Glaucoma is responsible for 10% of global blindness cases [57].

At the end stage of glaucoma, optic nerve damage is irreversible, often leading to permanent vision loss. Increased intraocular pressure (IOP) is a significant factor in glaucoma. Elevated IOP due to ocular hypertension can cause glaucomatous changes and progressive optic nerve damage. Glaucoma’s causes include idiopathic factors, hyperopia, trauma, cataracts, diabetes, uveitis, retinal vascular diseases, and intraocular surgeries. Recent studies suggest that prolonged smartphone use can lead to ocular hypertension, which may contribute to glaucoma, visual field loss, and eventual blindness [58].

Lowering IOP is critical for preventing vision loss. IOP is maintained by the balance between the production and outflow of aqueous humor within the eye. Current treatments for glaucoma include medications such as carbonic anhydrase inhibitors, alpha-2 adrenergic agonists, beta-blockers, and prostaglandin analogs, which either reduce aqueous humor production or enhance outflow. However, these medications may cause adverse effects such as bradycardia, low blood pressure, asthma attacks, or kidney stones [59]. Invasive treatments, including laser or surgical therapies, may also lead to complications such as re-bleeding or high failure rates.

In Experiment 2, we observed that the IOP-lowering effects of cassiae tea were comparable to those of Alphagan-P^®^ starting from the 4th month. Alphagan-P^®^ effectively reduces IOP by decreasing aqueous humor production and increasing uveoscleral outflow, making it a potent first-line treatment for glaucoma and ocular hypertension. However, its side effects, including red eyes, dry mouth, and fatigue, limit its use. Cassiae tea, in contrast, showed no adverse effects.

Our previous studies revealed that cassiae semen effectively reduces IOP due to its diuretic properties [60]. Compounds such as chrysophanol promote urination, while physcion inhibits 15-hydroxyprostaglandin dehydrogenase, enhancing vasodilation in the glomeruli’s afferent arterioles to facilitate urine output [61,62]. Increased urination reduces blood pressure, which may explain cassiae tea’s simultaneous IOP-lowering effects [63,64].

Additionally, acetylcholine (Ach) in cassiae tea enhances accommodative ability, helping to open Schlemm’s canal and the trabecular meshwork, thereby facilitating aqueous humor drainage and further reducing IOP [65].

Glaucoma and presbyopia often coexist due to shared pathophysiological factors, including dysfunction of the ciliary muscle, zonules lens, and crystalline lens. Accommodation, particularly during smartphone use, may exacerbate impaired aqueous outflow and central vitreous compression, resulting in IOP elevation and glaucomatous optic neuropathy [66].

Understanding the mechanisms underlying presbyopia is crucial for improving middle-aged individuals’ vision. On the basis of our findings, we suggest that cassiae tea, as a leisure activity, can help alleviate glaucoma and ocular hypertension, particularly in adults.

### 4.3. Presbyopia and Accommodation for Near Vision

Presbyopia typically begins to manifest between the ages of 40 and 45, as individuals experience increasing difficulty focusing on near and intermediate distances. By the age of 65, the eye becomes fully presbyopic, unable to focus on near images at all. In North America, Europe, and Japan alone, over 400 million people are affected by presbyopia for nearly half their lives, a prevalence that continues to rise due to population aging [67].

The mechanism of presbyopia involves the contraction of ciliary muscles to adjust the distance between the eyes and nearby objects, a process known as “accommodation”. However, the gradual and continuous loss of accommodative ability throughout the aging process leads to visual deterioration. For instance, the ciliary muscles undergo compensatory hypertrophy, resulting in difficulty contracting the ciliary fibers for accommodation [68]. Additionally, lenticular sclerosis develops with age due to the accumulation of damaged collagen fibers, which further impairs accommodation. By age 70, accommodation decreases to nearly zero, often necessitating presbyopic eyeglasses, especially as cataracts worsen.

Aside from aging, the overuse of smartphones has also been linked to premature presbyopia, attracting significant attention. Excessive smartphone use has been shown to impair near vision and accelerate the progression of presbyopia. Alarmingly, even teenagers who are heavily immersed in smartphone usage may experience poor accommodation and progressive myopia [69]. Addressing and improving both presbyopia and smartphone-induced presbyopia is a growing public health challenge.

Current treatment options for presbyopia include corrective eyeglasses (single-vision, bifocal, trifocal, or progressive addition lenses), refractive surgeries, or lens implants. These lens implants may involve fixed- or variable-focus systems, as well as surgical procedures aimed at restoring active accommodation to improve visual function. Eyeglasses remain the most accessible solution, but no available lenses can fully restore the range of accommodation. Single-vision glasses are limited to one distance, and multifocal lenses may restrict optical zones, potentially interfering with tasks such as driving or workplace activities. Surgical interventions carry inherent risks, including complications that may irreparably damage tissues. Meanwhile, recently developed pharmacological treatments may cause side effects and discomfort [70].

**Figure 4:**
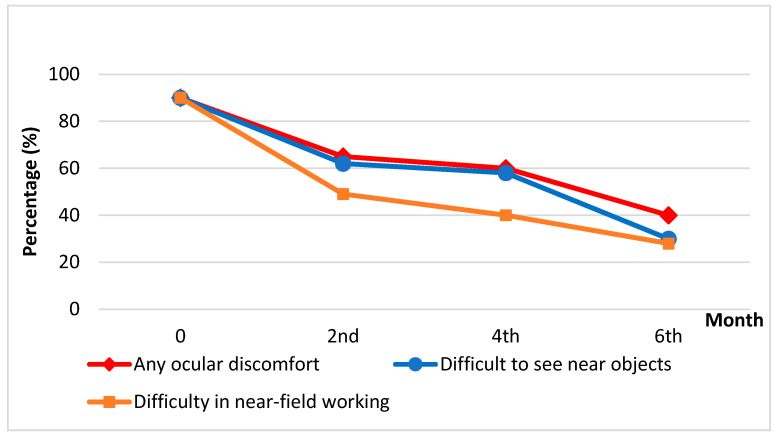
At baseline, approximately 85% of participants reported symptoms of ocular discomfort, difficulty seeing near objects, and challenges with near-field tasks. After consuming cassiae tea, these issues improved significantly. By the 6th month, the percentage of inconveniences caused by presbyopia had decreased to around 30–40%.

**Figure 5:**
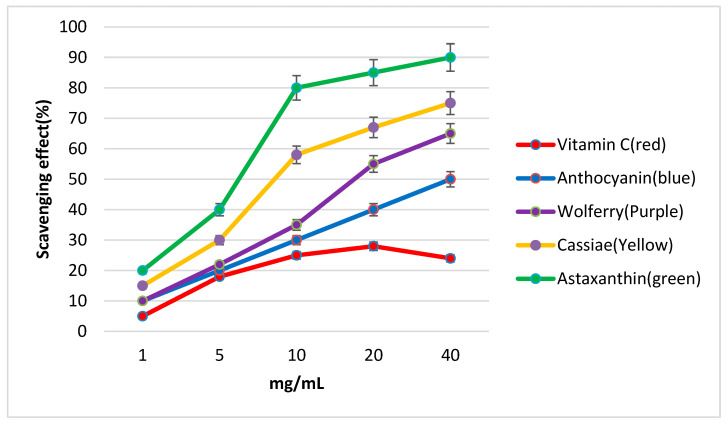
The antioxidant activities of various bioactive compounds and Chinese herbs were compared. Following a series of evaluations, the free radical scavenging effects were ranked as follows: astaxanthin (green line) > cassiae semen (yellow line) > wolfberry (purple line) > anthocyanin (blue line) > vitamin C (red line, control).

In Experiment 4, we observed that drinking cassiae tea improved near vision after four months. Experiment 5 further demonstrated that adequate doses of cassiae tea intake (the medium-dose group and high-dose group) significantly enhanced near vision, revealing a dose-dependent effect. In other words, the more cassiae tea consumed, the greater the improvement in near eyesight. Additionally, Experiment 6 (Figure 4) showed that presbyopic symptoms, such as ocular discomfort, decreased following cassiae tea intake. The percentage of individuals reporting difficulties seeing near objects and performing near-field tasks also declined. On the basis of these findings, we suggest that cassiae tea is an effective, user-friendly, and non-invasive option to alleviate presbyopia, improve quality of life, and reduce near vision impairment.

### 4.4. Pupil-Associated Problems

The pupil plays a critical role in regulating the amount of light entering the eye, allowing clear images of objects to form on the retina. Pupil size varies due to several factors, including circadian rhythms, light intensity, drug reactions, neurological disorders, mood, exercise, and ocular conditions such as glaucoma. In adults, normal pupil size ranges from 2 to 4 mm in bright light and 4 to 8 mm in the dark. Pupil size is controlled by the activities of two muscles: the sphincter and the iris dilator muscles.

Certain components in cassiae tea have been shown to increase serum acetylcholine (Ach) levels, which help contract the ciliary muscle, reduce refractive errors, and induce pupil constriction [71]. In Experiment 3, participants who drank cassiae tea experienced a decrease in pupil diameter, consistent with the findings of Mathôt et al., who demonstrated that smaller pupils improve visual sharpness [72].

Smaller pupils create a pinhole effect, which provides several visual benefits. For instance, smaller pupils block peripheral rays, reducing the effects of spherical aberrations under bright conditions. This effect also benefits individuals with myopia who have undergone refractive surgeries (e.g., LASIK), as it minimizes halos around lights at night [73]. Furthermore, smaller pupils reduce disturbances caused by secondary astigmatism or aberrations from corneal surgeries. Many studies have shown that smaller pupil sizes result in less image defocus and fewer aberrations, improving visual quality overall.

A smaller pupil also enhances the depth of field (DoF)—the range of distances within which objects are in acceptable focus. Bernal-Molina et al. demonstrated that individuals with larger DoF due to smaller pupils enjoy improved sharpness and visual acuity [74]. Ophthalmologists have confirmed that smaller pupils enhance far vision, DoF, and ocular accommodation while reducing chromatic aberrations. Sergienko et al. also reported that smaller pupils (particularly <3.0 mm) benefit individuals with presbyopia and myopia [75].

The advantages of smaller pupils extend further. Westheimer found that smaller pupils improve the resolution of high spatial frequencies, enhancing the ability to discern fine details [76]. Smaller pupils also reduce total response time to light, protecting against cataracts, retinopathies, and neuropathies caused by UV light stimulation. Conversely, overly large pupils may lead to glare, harsh shadows, and visual discomfort.

The relationship between smartphones and pupil response is also significant. Smaller pupils may benefit smartphone users by improving screen visibility through various mechanisms [77]. Therefore, we believe that the pinhole effect induced by cassiae tea consumption is particularly beneficial for individuals with presbyopia and frequent smartphone users.

### 4.5. SOD from Cassiae Semen

The elevation of superoxide dismutase (SOD) levels by cassiae semen has been reported in various studies. For example, extracts of cassiae semen significantly increase SOD and glutathione (GSH) levels, while reducing malondialdehyde (MDA), an end-product of lipid peroxidation often used as a biomarker for oxidative stress [78]. Sustained higher serum MDA levels are associated with cellular dysfunction, including in ocular structures. SOD, as a key antioxidant defense enzyme, plays a crucial role in cells exposed to oxidative stress.

In Experiment 7, our human study findings aligned with Kang’s animal research [79]. We demonstrated that SOD activity increased significantly (17.5 ± 2.8 U/mL) by the 4th month of cassiae tea intake and further increased to 19.3 ± 2.3 U/mL by the 6th month. SOD catalyzes the dismutation of superoxide anions into hydrogen peroxide (H2O2), which is subsequently detoxified into oxygen and water by catalase or GSH peroxidase. Elevated SOD levels can mitigate oxidative stress by neutralizing superoxide radicals, reducing toxic effects, and potentially treating various diseases.

Oxidative stress and its related ocular diseases may be managed by increasing SOD levels. Oxygen-derived free radicals are generated through normal metabolic pathways but can damage cellular components when antioxidant defenses are overwhelmed. This can lead to cell injuries and contribute to various ocular conditions. For instance: 1. Free radicals may damage ciliary muscle cells, elastic fibers, and the crystalline lens, leading to reduced accommodation and exacerbation of presbyopia. 2. Oxidative stress-induced dysfunction of trabecular meshwork cells in the cornea-iris angle may obstruct aqueous humor outflow, increasing intraocular pressure (IOP) and advancing glaucoma. 3. Environmental factors such as pollutants, UV radiation, and ozone can induce oxidative stress, leading to ocular surface inflammation and severe dry eye [80].

Additionally, oxidative stress can influence pupil size [81]. On the basis of these findings, we suggest that cassiae tea improves dry eye, lowers IOP, and enhances near vision through pupil constriction and increased SOD levels.

### 4.6. Comparison of Antioxidant Activities Between Cassiae Semen and Bioactive Compounds

Anthocyanins, a group of polyphenolic flavonoids found in fruits and vegetables, contribute to lipid metabolism regulation, body weight control, platelet activity modulation, and stroke risk reduction. Specific derivatives, such as cyanidin, pelargonidin, and cyanidin-3-glucoside (C3G), exhibit potent reactive oxygen species (ROS) scavenging abilities. In ophthalmology, C3G accelerates rhodopsin regeneration following light absorption, ameliorates ocular fatigue, enhances contrast sensitivity, and improves dry eye symptoms through SOD and GSH peroxidase activity [82,83].

Astaxanthin, a red-orange carotenoid found in aquatic organisms such as shrimp, crab, and krill, provides multiple health benefits, including treating renal diseases, gastrointestinal disorders, and atherosclerosis. It is particularly beneficial for various ocular conditions, such as dry eye, keratitis, cataracts, diabetic retinopathy, macular degeneration, high IOP, and optic nerve-related diseases, by increasing catalase, SOD, and GSH peroxidase activity [84]. Studies suggest that astaxanthin possesses superior antioxidant activity compared to other carotenoids like lutein, zeaxanthin, and α-carotene [85].

Wolfberry (goji berry) has been consumed throughout Asia for its nutritional and medicinal properties, including anti-aging effects, immune enhancement, and metabolic regulation. It also benefits eye health, helping manage dry eye, glaucoma, diabetic retinopathy, and night blindness. Amagase et al. demonstrated that consuming 120 mL/day of goji berry juice for 30 days improved SOD levels by 8.4% and reduced MDA levels by 8.7% in serum [86,87].

Vitamin C, a chain-breaking antioxidant, protects cells from oxidative stress-induced damage and cell death. Found in fruits (e.g., oranges, kiwifruit, wolfberry) and vegetables (e.g., broccoli, cabbage), vitamin C scavenges free radicals via the double bonds in its lactone ring. It has been shown to reduce the risks of oxidative stress-related ocular diseases, including cataracts, glaucoma, dry eye, presbyopia, and macular degeneration [88].

In Experiment 8, we compared the antioxidant capacities of various bioactive compounds and Chinese herbs using the DPPH assay. The results revealed that cassiae semen exhibited significantly better free radical scavenging activity than vitamin C, anthocyanins, and wolfberry, and was only slightly less effective than astaxanthin (Figure 3). Thus, we propose that cassiae tea possesses strong antioxidant properties that could be beneficial for ocular diseases such as dry eye, ocular hypertension, glaucoma, and presbyopia. Over 700 chemical compounds have been extracted from cassiae semen, with anthraquinones, naphthopyrones, and volatile oils identified as major active ingredients, likely contributing to its superior antioxidant effects [89].

### 4.7. Safety and Toxicity

Cassiae semen is widely used in both medicine and food, especially in China, Japan, and Korea. Many individuals consume cassiae tea daily as a beverage. Historical records in ancient medical texts describe cassiae semen as non-toxic and safe, with minimal side effects even with excessive or improper use [90]. Occasionally, mild diarrhea may occur after consuming non-roasted or non-baked cassiae semen.

In our experiments, no participants reported discomfort after six months of cassiae tea intake. Consequently, we conclude that cassiae tea is a safe and well-tolerated option for daily consumption, even for children.

### 4.8. Limitations of This Study

This study has several limitations:

Accuracy of IOP Measurements: We used a non-contact tonometer to measure IOP. While this method offers advantages such as rapid screening, ease of use, and infection prevention, it often overestimates IOP by 1–2 mmHg compared with other techniques [91,92]. As all measurement methods have inherent limitations, the absolute accuracy of IOP values remains uncertain. Nonetheless, precise IOP values were not the primary focus of this study.

Optimal Dosage of Cassiae Tea: The dosage of cassiae tea used in this study was based on the prescription from Dong et al. [15]. However, the exact dose required to achieve maximal pharmacological effects on near vision warrants further investigation. We plan to conduct a series of studies to explore this aspect in greater detail.

## 5. Conclusions

The prevalence of presbyopia has been exacerbated by widespread smartphone use and overdependence, as highlighted by Kim et al. [93]. To the best of our knowledge, this is the first study to quantitatively and qualitatively analyze the effects of daily cassiae tea consumption on presbyopia, dry eye, ocular hypertension, glaucoma, and poor accommodation.

Our findings suggest that regular dietary consumption of cassiae tea significantly benefits individuals with presbyopia and those engaged in near-distance work, such as smartphone users. Cassiae tea is a safe, comfortable, non-invasive, and affordable treatment option for managing presbyopia in daily life. Moreover, it may help slow the progression of presbyopia, including smartphone-induced presbyopia, thereby promoting overall ocular health.

## Figures and Tables

**Table 1:** Changes in Schirmer’s test scores after drinking cassiae tea.

	Time	Baseline	2nd Month	4th Month	6th Month
Group					
Control		3.1 ± 0.4	4.5 ± 1.4	3.9 ± 0.8	3.5 ± 0.9
Test group		3.5 ± 0.8	6.5 ± 0.7	15.2 ± 0.8 *	17.6 ± 0.9 *

Schirmer’s test scores at the 2nd, 4th, and 6th months were compared to baseline. * *p* < 0.05. The *p*-values for the test group were 0.02 at the 4th month (15.2 ± 0.8) and 0.01 at the 6th month (17.6 ± 0.9), respectively. Additionally, the unit of Schirmer’s test score was mm/5 min.

**Table 2:** Intraocular pressure (IOP) values following cassiae tea intake.

	Time	Baseline	2nd Month	4th Month	6th Month
Group					
Control		30.5 ± 0.5	30.2 ± 1.4	29.5 ± 0.8	29.5 ± 1.8
Test group		31.2 ± 0.8	26.5 ± 0.7	21.5 ± 2.1 *	18.5 ± 1.6 *
Eyedrop group		31.8 ± 1.5	25.8 ± 1.8	20.5 ± 1.9 *	17.5 ± 0.9 *

Group: vitamin C in water; test group: 2 cups/day of cassiae tea; eyedrops group: topical use of alphagan^®^ IOP values in the 2nd month, the 4nd month, and the 6th month were assessed compared with baseline. * *p* < 0.05. The *p*-values for the test group were 0.03 at the 4th month (21.5 ± 2.1) and 0.01 at the 6th month (18.6 ± 1.6). Similarly, the *p*-values for the eyedrop group were 0.0 at the 4th month (20.5 ± 1.9) and 0.009 at the 6th month (17.6 ± 0.9). The unit of IOP was mmHg.

**Table 3:** Measured pupil sizes after consuming cassiae tea.

	Time	Baseline	2nd Month	4th Month	6th Month
Group					
Control		3.0 ± 0.4	2.9 ± 1.4	3.1 ± 0.8	3.0 ± 0.9
Test group		3.2 ± 0.5	3.1 ± 0.5	2.4 ± 0.4 *	2.3 ± 0.3 *

Pupil sizes at the 2nd month, 4th month, and 6th month were compared to the baseline (* *p* < 0.05). Additionally, the unit of measurement for human pupil size was “mm”. The *p*-values for the test group were 0.03 in the 4th month (21.5 ± 2.1) and 0.01 in the 6th month (18.6 ± 1.6), respectively.

**Table 4:** Measuring near vision after cassia tea supplementation.

	Time	Baseline	2nd Month	4th Month	6th Month
Group					
Control		5.2 ± 0.8	4.9 ± 1.4	5.2 ± 0.9	5.1 ± 0.7
Test group		5.4 ± 1.0	5.1 ± 0.5	3.3 ± 1.2 *	2.5 ± 0.8 *

Near vision was analyzed at the 2nd, 4th, and 6th months, using baseline measurements for comparison (* *p* < 0.05). Near vision measurements were expressed in “Jaeger” units (J). The *p*-values for the test group were 0.02 in the 4th month (3.3 ± 1.2) and 0.01 in the 6th month (2.5 ± 0.8), respectively.

**Table 5:** Assessment of near vision in individuals consuming different doses of cassiae tea.

	Time	Baseline	2nd Month	4th Month	6th Month
Group					
Control		5.3 ± 1.2	4.9 ± 1.3	5.1 ± 0.8	5.3 ± 1.1
LDG		5.4 ± 1.0	4.8 ± 0.7	4.2 ± 1.1	4.1 ± 0.7
MDG		5.3 ± 1.1	5.0 ± 0.9	3.5 ± 1.3 *#	2.7 ± 1.2 *#
HDG		5.1 ± 1.0	4.7 ± 1.1	3.0 ± 1.2 *#	2.3 ± 1.0 *#

Control: Vitamin C in water; LDG: 1 cup/day of cassiae tea; MDG: 2 cups/day of cassiae tea; HDG: 3 cups/day of cassiae tea. In the first comparison, AA levels at the 2nd month, 4th month, and 6th month were assessed relative to baseline. In the second comparison, the near vision of the test groups (LDG, MDG, HDG) at each time point (2nd month, 4th month, and 6th month) was compared to the control group. The *p*-values for MDG were 0.04 in the 4th month (3.5 ± 1.3) and 0.03 in the 6th month (2.7 ± 1.2), respectively. The *p*-values for HDG were 0.03 in the 4th month (3.0 ± 1.2) and 0.01 in the 6th month (2.3 ± 1.0), respectively. * *p* < 0.05 in the first comparison, and # *p* < 0.05 in the second comparison. Additionally, near vision was measured in units of “J”.

**Table 6:** Changes in serum superoxide dismutase (SOD) levels following cassiae tea supplementation.

	Time	Baseline	2nd Month	4th Month	6th Month
Group					
Control		10.4 ± 1.8	11.2 ± 0.9	10.9 ± 2.7	11.5 ± 2.2
Test group		10.5 ± 2.3	10.8 ± 1.7	17.5 ± 2.8 *	19.3 ± 2.3 *

Serum SOD levels at the 2nd, 4th, and 6th months were compared to baseline. * *p* < 0.05. The *p*-values for the test group were 0.03 in the 4th month (17.5 ± 2.8) and 0.02 in the 6th month (19.3 ± 2.3), respectively. SOD values are expressed in units of U/mL.

## Data Availability

The data that support the findings of this study are available from the corresponding author upon reasonable request.
